# Neuroprotection in Glaucoma: Old and New Promising Treatments

**DOI:** 10.1155/2017/4320408

**Published:** 2017-10-17

**Authors:** Dario Rusciano, Salvatore Pezzino, Maria Giulia Mutolo, Rossella Giannotti, Aloisa Librando, Nicola Pescosolido

**Affiliations:** ^1^Sooft Italia S.p.A., Via Salvatore Quasimodo 136, Rome, Italy; ^2^Bioos Italia S.r.l., Viale Andrea Doria 21, Catania, Italy; ^3^Ophthalmology Clinic of the Sant'Andrea Hospital of Rome, Sapienza University of Rome, Rome, Italy; ^4^Department of Sense Organs, Sapienza University of Rome, Rome, Italy; ^5^Department of Cardiovascular, Respiratory, Nephrology, Anesthesiology and Geriatric Sciences, Sapienza University of Rome, Rome, Italy

## Abstract

Glaucoma is a major global cause of blindness, but the molecular mechanisms responsible for the neurodegenerative damage are not clear. Undoubtedly, the high intraocular pressure (IOP) and the secondary ischemic and mechanical damage of the optic nerve have a crucial role in retinal ganglion cell (RGC) death. Several studies specifically analyzed the events that lead to nerve fiber layer thinning, showing the importance of both intra- and extracellular factors. In parallel, many neuroprotective substances have been tested for their efficacy and safety in hindering the negative effects that lead to RGC death. New formulations of these compounds, also suitable for chronic oral administration, are likely to be used in clinical practice in the future along with conventional therapies, in order to control the progression of the visual impairment due to primary open-angle glaucoma (POAG). This review illustrates some of these old and new promising agents for the adjuvant treatment of POAG, with particular emphasis on forskolin and melatonin.

## 1. Introduction

Glaucoma is a worldwide leading cause of irreversible blindness. It is a multifactorial optic neuropathy characterized by the progressive loss of retinal ganglion cells (RGCs) and their axons [1].

Although the etiopathogenesis of glaucoma is not fully understood, high intraocular pressure (IOP) appears to be related to RGC death, both in the case of acute closed-angle glaucoma, when there is a sudden increase of IOP, and in the case of primary open-angle glaucoma (POAG), which develops relatively slowly over the years. POAG can be hypertensive (IOP > 21 mmHg) or normotensive (NTG: IOP < 21 mmHg). In either case, medical or surgical lowering of the IOP appears to delay the disease's progression [2, 3].

In glaucomatous patients, the increased resistance to aqueous drainage through the trabecular meshwork (POAG) and the obstruction of the drainage pathway by the iris (primary closed-angle glaucoma) are the main causes of IOP elevation [4, 5]. Furthermore, several risk factors have been associated with glaucoma pathogenesis: older age, black race, Hispanic origin, family history of glaucoma, myopia, diabetes mellitus, and use of systemic or topical corticosteroids [6, 7].

**Figure 1:**
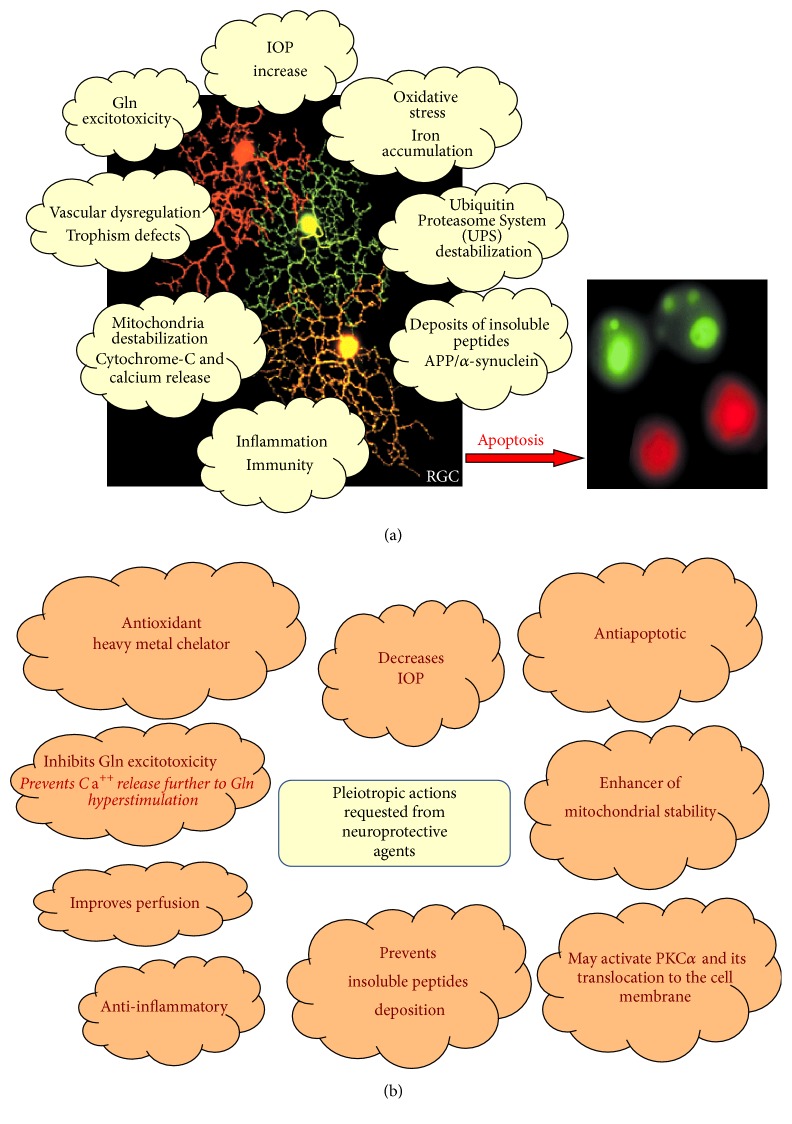
(a) Several different factors may contribute to the development and progression of POAG, finally triggering cell death, usually by apoptosis, of RGC. These factors could be considered potential targets of neuroprotective agents. (b) Due to the multifactorial nature of POAG, pleiotropic effects are expected from neuroprotective agents in order to achieve efficient neuroprotection.

Therefore, with POAG being a complex multifactorial disease, several events converge to induce RGC loss, such as IOP elevation, ischemia/reperfusion damage, oxidative/nitrosative stress, neurotrophic growth factor deprivation, activation of autoimmunity, and glutamate neurotoxicity (Figure 1(a)).

The growing knowledge of this disease and its etiopathogenesis has prompted the study of new targets and therapeutic agents aimed at stopping or delaying RGC neurodegeneration (Figure 1(b)). The goal of the present review is to discuss some of the old and promising new agents that may contribute to a better treatment of POAG.

## 2. RGC Death and Neuroprotection Targets

POAG presents with a typical progressive visual field loss and optic disk cupping due to the massive death of RGCs and their nerve fibers (usually more than 50% of RGCs are dead when the first visual field abnormalities are detectable) [8].

The major risk factors linked to the starting of the chain of events finally leading to overt POAG may be of genetic, mechanical, biochemical, or hemorheological nature. None of them by itself can explain or predict the insurgence and progression of the disease, which is most likely controlled by a combination of different factors, some of which probably still need to be identified.

A certain degree of familiarity exists for POAG, which has led to the discovery of several genes that may be associated with the disease [9]; however, no single gene by itself has been found to cause POAG.

IOP increase is the main mechanical risk factor, which may stress both retinal layers and the* lamina cribrosa* through which RGC nerve fibers leave the eye, forming the optic nerve. This may result in ischemic injury, glial cell activation, reduced axoplasm flow, and neurotrophin deprivation, finally causing RGC apoptosis [10]. Further glutamate release from dying cells may cause an excitotoxic response in neighboring RGCs, triggering their apoptotic death in a vicious circle [11].

Oxidative stress induced by oxygen and nitrogen reactive species is also implicated in the etiology of POAG and several other eye disorders [12–15]. Several mechanisms involving antioxidant enzymes such as superoxide dismutase (SOD), as well as antioxidants such as glutathione and ascorbate, protect the eye from oxidative stress [16]. It has been found that POAG patients exhibit low levels of circulating glutathione, suggesting a general impairment of the antioxidative defense [17], while reduced expression of the antioxidant enzymes SOD and glutathione S-transferases was found in the aqueous humor of POAG patients, suggesting that this state could aggravate the balance between both oxygen- and nitrogen-derived free radical production and their detoxification.

Defective blood perfusion of the retina and the optic nerve head may also be critical factors in POAG development and progression, with more relevance in the case of NTG [18].

**Figure 2:**
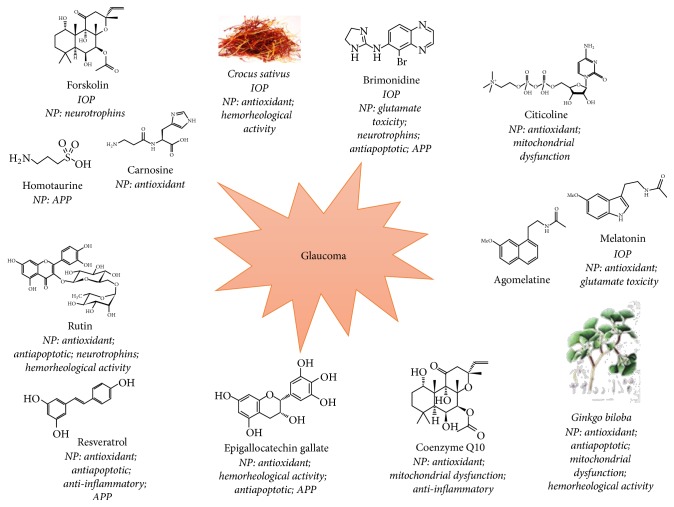
Schematic rendering of neuroprotective agents described in this review, summarizing their neuroprotective activities: no single molecule is endowed with all the required activities; brimonidine shows the highest number (5). IOP:* hypotonizing* effects; NP: neuroprotection mechanisms; APP (amyloid plaque protein): decrease of APP pathologic processing.

Despite the identification of several risk factors for POAG, the main—if not the only—therapeutic target to treat glaucoma still remains the IOP. Even though clinical studies have shown that decreasing the IOP is necessary, it is not the only condition capable of* preventing* glaucoma development or progression [4]. Therefore, neuroprotection strategies have been developed in recent years, not to replace but to complement the classical IOP lowering approach [19], aiming at slowing down POAG progression. Several targets have been proposed for neuroprotection, and molecules acting on such targets can be classified as oligopotent or multipotent, depending on whether they may act on multiple targets or not. We have chosen here a selection of molecules belonging to either class, mostly based on our direct experience with them (Figure 2).

## 3. Forskolin

**Figure 3:**
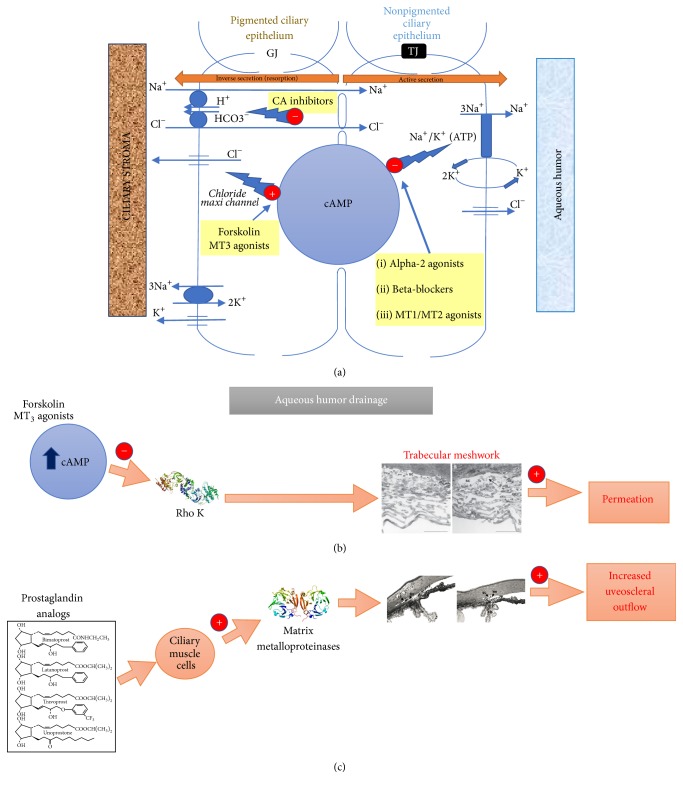
Regulation of IOP by different classes of molecules and mediators. (a) Central role of cAMP in the regulation of neat AH production. A decrease of cAMP in NPE ciliary cells appears to lead to a decreased efflux through the ATP-dependent Na+/K+ channel; an increase of ATP might trigger the activation of the chloride maxi channel in PE ciliary cells, leading to “inverse secretion” towards the stroma, finally reducing the AH influx into the anterior chamber. CA inhibitors work independently of cAMP and decrease carbonate exchange with the stroma, finally reducing chloride transit to the anterior chamber and water secretion. GJ: gap junctions; TJ: tight junctions; MT1,2,3: melatonin receptors; CA: carbonic anhydrase. (b) An increase of cAMP induced by forskolin or melatonin through the MT3 receptors may lead to inhibition of the Rho kinase, which in turn results in the disorganization of the TM cells cytoskeleton and finally to an increased TM outflow. (c) Prostaglandin analogs upregulate MMPs (matrix metalloproteases) which degrade extracellular proteins (mainly collagen) in the uveoscleral pathway, thus increasing AH outflow through this way.

Forskolin is a diterpene isolated from the root extract of* Coleus forskohlii *species [20]. It is a receptor-independent activator of adenylate cyclase. In forskolin-treated cells, the intracellular concentration of the second messenger cAMP is rapidly increased [21]. The adenylate cyclase complex is present both in ciliary body epithelial cells [22], deputed at producing the aqueous humor (AH), and in trabecular meshwork (TM) cells, regulating the aqueous humor outflow [23]. Numerous studies have shown that forskolin is able to reduce IOP in animals and humans [24–32]. This happens probably because cAMP elevation in the ciliary body may lead to the activation of the chloride maxi channel in pigmented epithelial cells, facing the stroma, thus leading to resorption of AH from the posterior chamber into the stroma (Figure 3(a)) [33]. Conversely, alpha-agonists and beta-blockers decrease cAMP production in nonpigmented epithelial cells, thus decreasing the activity of the chloride channels facing the posterior chamber, leading to decreased AH secretion (Figure 3(a)) [34]. Moreover, cAMP elevation in TM cells might trigger the disassembly of the actin cytoskeleton through PKA activation and Rho kinase inhibition [35], thus increasing the TM outflow (Figure 3(b)) [36]. Therefore, forskolin treatment results in a reduced amount of AH accumulation in the anterior chamber (decreased secretion and increased outflow) in response to adenyl cyclase activation [27, 37]. Interestingly, the reduction of IOP by forskolin occurs through mechanisms that are not fully exploited by the existing hypotonizing glaucoma drugs. In fact, glaucoma patients, in whom the target pressure could not be reached even by the combination of three or four hypotonizing drugs, experienced a further decrease of their IOP when* oral* forskolin was added to their therapy [38]. Therefore, forskolin may have* indirect* beneficial neuroprotective effects on RGC by reduction of the IOP.

Beyond its action on IOP, forskolin may exert* direct* neuroprotection through different mechanisms. Neurotrophins, such as NGF and BDNF, have been found to be decreased in glaucoma [39–41]. In a rat model of experimental glaucoma, artificial elevation of IOP cripples the optic nerve head at the level of the lamina cribrosa, swelling nerve fibers and blocking the axonal flow of BDNF, while expression of the cognate receptor TrkB is increased at the optic nerve head [42]. Similar findings have also been reported in a spontaneous glaucoma model in the American Cocker Spaniel dog [43]. In this respect, forskolin could provide some degree of direct neuroprotection through the activation of paracrine signaling, since it has been shown to induce BDNF expression by astrocytes and vascular endothelial cells [44, 45] and to promote translocation of the cognate receptor TrkB to the neuron cell membrane [46]. Moreover, forskolin has been shown to be necessary for neuron cell survival* in vitro* [47, 48]. Also, optic nerve regeneration that is promoted by oncomodulin needs cAMP elevation (as it can be promoted by forskolin) in order to work efficiently [49–51]. Finally, elevation of cAMP levels is known to reduce excitotoxic damage and to inhibit the resulting apoptotic cell death [52]. Forskolin has been shown to protect neuronal cell cultures from soman and sarin, which are toxic organophosphate chemicals [53], and to attenuate the adverse effects of long-term Schwann cell denervation on peripheral nerve regeneration* in vivo *[54].

From a clinical perspective, it was shown that oral treatment of POAG patients with a food supplement based on forskolin, besides decreasing the IOP (as expected), also improved their PERG amplitude, thus suggesting a positive effect on RGC survival and/or function [55].

## 4. Forskolin with Homotaurine and L-Carnosine

Recent data have shown an association between POAG and Alzheimer's disease [56], and the toxic accumulation of insoluble beta-amyloids may also occur in RGCs [57, 58].

Homotaurine has been reported to decrease the accumulation of amyloid plaques in neurons [59], and carnosine has shown extensive neuroprotective efficacy in a rat model of experimental glaucoma [60]. Therefore, based on these results, the synergic neuroprotective effect of forskolin, homotaurine, and L-carnosine has been investigated in a rat model of experimental glaucoma [61]. After the induction of retinal ischemia through the artificial increase of the IOP, forskolin, homotaurine, and L-carnosine have been injected in the rat's eye. Following the combined treatment, a synergic neuroprotective effect on RGC survival was reported, in association with the upregulation of the prosurvival pathway of PI3K/Akt [62] and the inhibition of the proapoptotic pathway linked to GSK-3*β* [63–65]. Such neuroprotective effect is also correlated with the reduction of calpain activity, known to be linked to neurodegenerative events [66, 67].

Support of the above comes from a recent clinical study [68] carried out on glaucomatous patients with IOP compensated by topical drugs, which evaluated the additional neuroprotective effect of the food supplement containing forskolin, homotaurine, carnosine, folic acid, vitamins B1, B2, and B6, and magnesium. Treatment with the food supplement resulted in a further significant decrease of the IOP (most likely due to forskolin) and an improvement of PERG amplitude and foveal sensitivity, parameters related to RGC function.

## 5. *Crocus sativus* (Saffron)

Saffron is derived from the pistils of* Crocus sativus*, a well-known traditional Chinese medicine [69], and contains high concentrations of the carotenoids crocin and crocetin. Multiple divalent carbon bonds in saffron compounds confer their powerful radical scavenging and antioxidative properties [70–72]. It is likely because of this antioxidant effect on a clogged trabecular meshwork that high dose oral saffron treatment may further decrease IOP in POAG patients already undergoing different hypotonizing treatments [73].

More recent studies have highlighted the neuroprotective properties of saffron. In a rat model of continuous blue light exposure, saffron dietary supplement protects photoreceptors from photooxidative damage, maintaining both morphology and function [74]. Similar results against light-induced damage were obtained in mice and were attributed to the inhibition of caspase activity [75].

The main saffron components of interest for their associated biological activity are the carotenoid derivatives crocetin and crocin [69]. In a model of rat brain cerebral contusion, crocetin's protective effects were related to its proangiogenic and antiapoptotic activities [76].

Ocular hypertension, as well as the consequent reduced blood flow into the eye circulation, is the basis for the longstanding ischemic hypothesis of glaucoma [77, 78]. Crocin improves both the retinal and the choroidal blood flow* in vivo* and consequently facilitates retinal function recovery following IOP increase [79]. Qi et al. have demonstrated that injection of crocin in the eye prevented RGC apoptosis after retinal ischemia/reperfusion injury by involving the prosurvival PI3K/AKT signaling pathway. Furthermore, they have found that crocin increases the Bcl-2/BAX ratio, which contributes to the antiapoptotic effect of this molecule [80].

## 6. Brimonidine

Brimonidine is an adrenergic alpha-2 receptor (*α*2r) agonist, currently used in glaucoma therapy. Agonist mediated activation of *α*2r leads to a decrease of cellular cAMP, which in turn results in decreased secretion of AH into the posterior chamber (Figure 3(a)) [34], thereby leading to a decreased IOP in the initial and long-term treatment of ocular hypertension and glaucoma [81–83].

Experimental studies have shown that brimonidine has also neuroprotective activity. In a rat model of axotomized eyes, intravitreal injection of brimonidine enhanced the survival and the electrophysiological activity of RGC, by activating the Trk-MAPK/ERK and Trk-PI3K signaling pathways [84]. Similarly, in a rat model of secondary neurodegeneration induced by partial crush of the optic nerve (thus independent of IOP elevation), the intraperitoneal administration of *α*2r agonists (brimonidine, clonidine, and a synthetic compound AGN 191103) has been shown to be neuroprotective, whereas timolol injection had no effect [85]. In a retinal ischemia model, the intraperitoneal administration of brimonidine prevented the accumulation of toxic concentrations of extracellular glutamate and aspartate and preserved the ERG-b wave [86]. Similarly, systemic treatment with brimonidine prevented the elevation of N-methyl-D-aspartate (NMDA) receptor expression in rat ischemic retinal injury induced by acute IOP elevation [87] and limited RGC death in both an isolated rat retina and an* in vivo* rabbit retinal excitotoxicity model, through the modulation of the NMDA receptor function [88].

Systemic administration of brimonidine has been shown to protect RGC in a rat model in which chronic ocular hypertension was induced by laser photocoagulation of the trabeculae [89].

Brimonidine has been shown to upregulate neurotrophic factors expression in the retina, such as fibroblast growth factor 2 (FGF2) and BDNF [90, 91]. The neuroprotective effects of brimonidine on RGC are also evident after topic ocular administration in adult rats [92].

Most recently, a new IOP independent neuroprotection mechanism has been identified for brimonidine and adrenergic *α*2r agonists, which appears to inhibit the amyloidogenic pathway that leads to the formation of neurotoxic amyloid plaques, by stimulating the alternative nonamyloidogenic enzymes [58].

Clinical studies have shown that topical brimonidine improved the visual outcome of patients undergoing laser treatment for classic extrafoveal or juxtafoveal choroidal neovascularization treatment [93] and that brimonidine, but not timolol, topical therapy, improved contrast sensitivity of glaucoma patients after 3 months of treatment [94].

More recently, a long-term clinical study has indicated that topical brimonidine treatment may indeed protect the RGC of glaucomatous patients. The clinical comparison between brimonidine and timolol in preserving the visual function of NTG patients over a period of 4 years of observation has shown that, despite an identical effect on IOP, after 2 years, those patients treated with brimonidine were less likely to have disease progression than those treated with timolol [95].

## 7. Cytidine-5′-diphosphocholine (Citicoline)

Citicoline is a naturally occurring cell endogenous compound, intermediate in the synthesis of membrane phospholipids such as phosphatidylcholine [96]. Experimental studies have shown that citicoline may indeed increase the synthesis of phospholipids in the CNS [97] and indicated a neuromodulator effect and a protective role of this molecule on RGC [98]. In rodent retinal cultures and animal models, citicoline triggered antiapoptotic effects, increased the retinal level of dopamine (one of the most important neurotransmitters involved in retinal and postretinal visual pathways) [99], and prevented the thinning of retinal nerve fiber layer [100]. However, whether dopamine itself works as a neuroprotectant for RGC is not clear yet, since no direct effects of dopamine on RGC survival have been reported.

Citicoline has been shown to protect the retina* in vivo *against kainate-induced neurotoxicity [101] and to rescue rat RGC following partial optic nerve crush [102].

A beneficial effect of citicoline oral supplement has been demonstrated in patients with nonarteritic ischemic optic neuropathy. At the end of the study, PERG, visual evoked potentials, and visual acuity were improved compared to pretreatment values and to a group of patients with no treatment during the same period [103].

Other clinical studies reported citicoline neurotrophic effects in POAG management [104–107]. The effect on the rate of progression of visual field loss (dB/year) in subjects receiving citicoline oral supplementation was evaluated in a multicenter study on patients with progressive glaucoma. Patients receiving citicoline for two years showed a reduction in the mean rate of progression from −1 dB/year to −0.15 (±0.3) dB/year at the end of the study [108].

In another recent clinical study on POAG patients, Parisi et al. have shown that topical treatment with citicoline induces an enhancement of the retinal bioelectrical response (increase of PERG amplitude) with a consequent improvement of the bioelectrical activity of the visual cortex (shortening and increase of VEP implicit time and amplitude, resp.) [109].

## 8. Melatonin and Agomelatine

Melatonin is a hormone ubiquitously distributed in living systems, from bacteria to plants and animals. In mammals, including humans, it is secreted during darkness by the pineal gland and inhibited by light, so that it can modulate the body's sleep pattern. The pineal gland is the main source of melatonin, although other organs and cells such as skin, gastrointestinal tract, platelets, and lymphocytes can also make it [110]. Vertebrates' and mammals' retinas have also been shown to be able to synthesize melatonin, although confirmatory data are still needed for primates' retina [111]. Melatonin receptors (MT1, MT2, and to a lesser extent MT3) are consequently found in many tissues [112], including the eye, where they are well represented in retinal cells [111] and the ciliary epithelium [113].

The lipophilic nature of melatonin allows it to easily cross the hematoencephalic and hematoretinal barriers, thus reaching all tissues and the eye with good efficiency in a short time [114]. Melatonin can affect tissue metabolism and survival via receptor-independent and receptor-dependent mechanisms. The main receptor-independent activity is due to its strong antioxidant potential. Melatonin is a potent free radical scavenger and antioxidant, different from the other typical antioxidants. In fact, melatonin and its metabolites are able to neutralize numerous toxic oxygen and nitrogen reactive species (ROS and NOS, resp.) with high efficiency: one melatonin molecule has the capacity to scavenge a large variety of ROS/NOS, up to 10 molecules, versus the classic antioxidants that scavenge one or less of them. Therefore, melatonin is a more potent antioxidant than vitamins E and C [115]. Moreover, the large spectrum antioxidant activity of melatonin is potentiated by its regulatory activity on endogenous antioxidant and prooxidant enzymes, upregulating the former and downregulating the latter [116]. These activities designate melatonin as a neuroprotective agent in several neurodegenerative diseases, in which oxidative damage to neurons is a major player [117]. The efficacy of melatonin in preventing neuronal cell death and ameliorating Parkinson's disease (PD) symptoms has been demonstrated in animal models of PD [118].

In the eye, melatonin has been shown to protect human retinal pigment epithelial cells against oxidative stress [119] and to slow down photoreceptor degeneration in a mouse model of retinitis pigmentosa [120]. Moreover, the suppression of melatonin subtype receptor MT1 has been shown to decrease the viability of photoreceptors and RGCs [121, 122].

Glutamate accumulation in extracellular spaces can be potentially neurotoxic to the retina [123], and the impairment of glutamate transporter expression precedes the depression of glutamine synthase activity during ocular pressure loading [122]. In the hamster retina, it has been shown that melatonin may increase glutamate uptake and glutamine synthase activity, thus decreasing glutamate neurotoxicity [124].

Melatonin and its analogs have shown hypotonizing effects in both experimental animal models and glaucomatous patients [125–127]. Significant reductions of retinal melatonin levels were found in the rat model of glaucoma induced by chronic ocular hypertension [128]. The localization of melatonin receptors in the iris and ciliary processes strongly suggests that they are indeed involved in IOP regulation [129, 130], most likely through a mechanism that involves the putative MT3 receptors and a local increase in cAMP [131], similar to what has been described before for forskolin (Figure 3(a)). Correspondingly, preliminary clinical observations indicate a cooperative effect on IOP reduction by melatonin and forskolin (Pescosolido, personal communication).

Hypoxia has also been involved in the development of glaucoma [132–134]. Melatonin has shown neuroprotective effects against hypoxia-induced retinal ganglion cell death in neonatal rats [135].

Impairment of ocular blood flow is also a relevant player in the etiopathogenesis and progression of the glaucomatous optic neuropathy [132]. IOP or blood pressure circadian fluctuations cause an unstable oxygen supply, triggering further damage to RGCs [136]. Melatonin might contribute to the attenuation of these events, both on the IOP and on the blood flow control sides, since it is known to have vasoactive properties and shown to modulate arterial vasoconstriction [137].

Recognizing its beneficial antioxidant and ocular hypotensive properties, several melatonin related compounds, such as the synthetic analogs and the specific agonists of melatonin receptors, are under investigation [138]. Among the melatonin analogs, agomelatine is currently attracting interest for its pharmacological activities [110, 118, 127, 139–141]. Agomelatine is a drug used in the treatment of major depressive disorders. It was developed as a nonselective MT1/MT2 agonist [142], and it was also revealed to be an antagonist of the 5-HT 2C serotonergic receptors [141, 143, 144].

Agomelatine has been shown to reduce rabbit's intraocular pressure under normotensive and hypertensive conditions [140]. In a recent clinical study, the hypotensive activity of oral agomelatine in eyes of POAG patients was revealed: agomelatine treatment resulted in a significant and stable hypotonizing effect after 15 and 30 days of treatment [127]. Its hypotonizing activity on IOP appears to follow its ability to activate both the MT2 and the MT3 receptors. In fact, specific antagonists to these receptors (4PPDOT and prazosin) could attenuate agomelatine effects on rabbits' IOP [140].

Agomelatine has also shown neuroprotective effects: it could decrease glutamate release, thus reducing its excitotoxic effect, in the rat hippocampus [145].* In vivo* treatment with agomelatine reduces the chronic cerebral hypoperfusion responsible for vascular dementia and limits cholinergic dysfunction, oxidative stress, and tissue damage in mice [146]. The neuroprotective effects of agomelatine and melatonin against NMDA-receptor-mediated white matter lesions have been shown in a newborn mouse experimental model. Mice that received intraperitoneal agomelatine or melatonin had significant reductions in size of white matter cysts induced by the glutamatergic analog, when compared with controls [139].

## 9. *Ginkgo biloba* Extract

*Ginkgo biloba* is a native tree of China with various uses in traditional medicine and also as a source of food [147]. The leaf extract from* Ginkgo biloba* (GBE) is rich in biologically active ingredients (mainly flavonoids and terpenoids), which can scavenge free radicals and protect cells from lipid peroxidation [148–151]. More interestingly, the polyphenolic flavonoids that are richly present in GBE can act as antioxidants at the mitochondrial level (where other antioxidants cannot work), stabilizing mitochondrial membranes and improving their energetic balance specifically in neuronal cells [152, 153]. This is an important contribution for glaucoma treatment, since mitochondrial dysfunction has been strongly implicated in POAG pathogenesis [154].

In several experimental studies, GBE has been shown to exert antioxidant and neuroprotective properties [155–161]. Elevated levels of nitric oxide contribute significantly to the pathogenesis of ocular diseases [162]. Nitric oxide reacts with superoxides to form peroxynitrites [163], which cause nitrosylation of cellular proteins, DNA, and lipids, ultimately leading to RGC death [164]. It was demonstrated* in vitro* that GBE can scavenge nitric oxide [165] and possibly inhibit its production [166].

The protective activity of GBE on isolated rat retinas was evaluated on rats orally treated (versus untreated controls) with the extract for 10 days. Upon a challenge of the isolated retinas with an oxidant perfusion, GBE contrasted the decrease of ERG-b wave amplitude due to the oxidative damage [167].

The unstable oxygen supply to the retina and the optic nerve caused by high IOP, blood pressure fluctuations, or disturbed autoregulation also leads to increased oxidative stress, a main contributor to glaucomatous damage [136]. Beside its antioxidant properties, GBE also shows hemorheological and vasoactive effects, promoting erythrocytes deformability, decreasing fibrinogen levels, and improving blood viscosity and viscoelasticity [168], and increases microcirculation by improving the endothelium-dependent vasodilation [169]. Consistently, clinical observation has shown that GBE was able to significantly increase diastolic and systolic velocity in the ophthalmic artery (OA) of healthy volunteers [170]. Another clinical study evaluated the effects of GBE in NTG patients, in which vascular dysregulation appears to play a critical role. In a 4-month crossover design in a group of 27 patients with bilateral visual field damage resulting from NTG, a significant improvement in visual field parameters was recorded in association with oral delivery of 40 mg GBE, three times daily, with no significant changes in IOP, blood pressure, or heart rate [171].

In another controlled clinical study on 52 POAG patients, those treated with GBE showed, after 3 months of treatment, a relevant decrease of endothelin-1 (ET1, responsible for peripheral vasoconstriction), resulting in increased flow-dependent vasodilation. This was paralleled by a decrease of malondialdehyde-modified low-density lipoproteins and plasma malondialdehyde levels, indicating the activation of an antioxidant response and the attenuation of oxidative stress [172].

Apoptotic cell death is a hallmark of POAG damage and has been shown at the level of the trabecular meshwork [173] and the RGC layer [174]. GBE also shows antiapoptotic properties. Pheochromocytoma cells (PC12) treated with GBE were protected from mitochondrial damage induced by serum deprivation or by staurosporine through mechanisms that result in attenuated release of cytochrome-C and less DNA fragmentation, while DNA microarray assay results indicate that transcription of multiple apoptosis-related genes is either up- or downregulated in cells treated with GBE [175]. Moreover, GBE effects on mitochondria-dependent caspase pathway in cardiomyocytes exposed for 24 hours to hypoxia and four hours to reoxygenation resulted in inhibition of cytochrome-C release from mitochondria, thus decreasing caspase-3 activity and the resulting apoptotic cell death [176]. Finally, in an experimental* in vivo *study, it was demonstrated that GBE inhibited the apoptosis of RGC in guinea pigs after optic nerve transection, thus protecting their morphology and function [177].

## 10. Coenzyme Q10

Another even more specific agent targeting mitochondria for neuroprotection is the coenzyme Q10 (CoQ10), which is an essential membrane cofactor, with a strong antioxidant activity, in the mitochondrial respiratory chain [178, 179]. It also appears to be able to modulate gene expression with anti-inflammatory effects [180]. CoQ10 has been suggested to have a beneficial role in several neurodegenerative diseases, like Alzheimer's disease, Parkinson's disease, Huntington's disease, chorea, and others, also including glaucoma [181]. In fact, in neurodegenerative diseases, external oxidative stress induces mitochondrial dysfunction, which in turn leads to the increase of ROS generation, and finally leads to apoptotic cell death of the neuronal cells [182]. CoQ10 has been shown to inhibit ROS generation, to maintain mitochondrial membrane potential during oxidative stress, and to reduce the amount of mitochondrial ROS generation in neuronal cell cultures [183]. Furthermore, the inhibition of oxidative stress by CoQ10 increases the mitochondrial mass and improves the bioenergetic function in primary optic nerve head rat astrocyte cultures [184].

Glutamate excitotoxicity and oxidative stress, besides IOP elevation, are known risk factors for POAG development and RGC death [185, 186]. High levels of glutamate have been found in the retina of animal models of glaucoma [187, 188]. Accordingly, it has been reported that CoQ10 protects retinal cells* in vitro *against oxidative stress induced by hydrogen peroxide and protects them* in vivo *after intravitreal injection of N-methyl-D-aspartate [189]. Intraocular administration of CoQ10 reduces the synaptic glutamate and delays apoptosis of rat RGC after retinal ischemia/reperfusion [190]. In a similar experiment, it was demonstrated that CoQ10 results in RGC protection after artificial elevation of extracellular glutamate [191]. Most recently, the role of oral CoQ10 supplement against the effects of glutamate excitotoxicity and oxidative stress on RGC degeneration has been addressed in the spontaneous DBA/2J mouse model of glaucoma [192]. After feeding the animals for six months with the supplement, results showed that CoQ10 preserved mitochondrial DNA content and the Tfam/OXPHOS complex IV protein expression in the retina of glaucomatous DBA/2J mice, triggering an improvement of RGC survival and morphological and functional preservation of the optic nerve head's axons.

Glaucoma is widely known to be associated with increased RGC apoptosis [193]. Caspase-7 plays a critical role in this process [194] since RGCs of mice knocked out for caspase-7 have been shown to be protected from apoptotic death [194]. More recent data also suggest an important role for Fas receptors and caspase-3-mediated apoptosis in the pathophysiology of glaucomatous neurodegeneration [195].

Along this line, the antiapoptotic activity of CoQ10 has been evaluated in a rat model of cultured RGCs exposed to external damage and in a mouse model of kainic acid-induced retinal damage. In these experimental models, CoQ10 significantly increased RGC viability by preventing caspase-3/7 activation [196].

Clinical studies also suggest that CoQ10 treatment may protect RGCs in human glaucoma. Topical administration of CoQ10 associated with vitamin E has been shown to positively affect retinal function in POAG patients. Patients treated with such association showed PERG improvement with consequent enhancement of the visual cortical responses [197].

Timolol is a nonselective beta-adrenergic receptor antagonist and is one of the main molecules indicated for glaucoma treatment. Unfortunately, in some cases, adverse cardiovascular effects can occur, and CoQ10 has been shown to be effective in reducing such systemic side effects induced by timolol [198, 199].

## 11. Polyphenols: Epigallocatechin Gallate, Resveratrol, and Rutin

Polyphenols are secondary plant metabolites generally synthesized from phenylalanine and used by plants in the defense against ultraviolet radiation or aggression by pathogens [200]. In the last decades, together with the realization that many pathologies, and aging itself, are caused by an excess of oxidative damage, there has been much attention to the health benefits of plant polyphenols (mainly those belonging to the class of flavonoids), due to their strong antioxidant properties [201].

## 12. Epigallocatechin Gallate

Catechins are flavanols, a subclass of flavonoids. They are the main components of green tea extract, among which epigallocatechin gallate (EGCG, also known as epigallocatechin-3-gallate) is the most abundant. Catechins may act as radical scavengers, iron chelators, and modulators of prosurvival genes expression and the PKC signaling pathway [202, 203]. More recent studies indicate that these properties of green tea catechins do not fully explain their neuroprotective capacity and, in fact, a wider spectrum of intracellular molecular targets may be implicated, such as the regulation of calcium homeostasis [204] and the activation of mitogen-activated protein kinase (MAPK) [205, 206], phase II antioxidant detoxifying enzymes [207], and serine/threonine protein kinase AKT [208]. Moreover, it has been shown that EGCG promotes the processing of amyloid precursor protein (APP) via the nontoxic *α*-secretase pathway [209] and reduces the formation of *β*-amyloid fibrils [210], which may be of particular relevance to both AD and glaucoma.

The neuroprotective effect of EGCG was demonstrated against the oxidative stress directly delivered to neural cells* in vitro* and* in vivo* in a mouse model of ischemia/reperfusion of the retina after artificial IOP elevation [211]. Along a similar line, it was shown that oral administration of EGCG protects RGCs from degeneration in a mouse model of chronic glaucoma obtained after microbeads injection in the anterior chamber [212] and in the optic nerve crush rat model [213]. Intravitreal injection of oxidants such as sodium nitroprusside (which generates NO spontaneously) triggers significant photoreceptor apoptosis with the rest of the retina relatively unaffected [214, 215]. When EGCG is injected into the rat eye together with sodium nitroprusside, its detrimental influence on retinal photoreceptors was attenuated [216].

The pharmacokinetics of EGCG have been addressed by HPLC analysis, showing that, after a single oral administration, EGCG is widely distributed in mouse tissues and reaches the central nervous system in a short time (6 h); a second administration after a 6 h interval enhances tissue levels four to six times above that of a single administration [217].

EGCG is nongenotoxic, even when administered to animals at doses that are significantly higher than those intended for humans [218].

Clinical efficacy of a short-term oral supplementation of EGCG has been studied by PERG analysis (addressing the electrical activity of RGC), showing that the treatment might favorably influence the inner retinal function in human eyes of glaucomatous patients with early to moderately advanced damage [219].

## 13. Resveratrol

Resveratrol belongs to a class of polyphenolic compounds called stilbenes. Some plants (notably red grapes) produce resveratrol and other stilbenoids in response to stress, injury, fungal infection, or ultraviolet (UV) radiation [220]. Besides its own antioxidant activity, more evident in the test tube than* in vivo* [221], resveratrol has been shown to induce several antioxidant enzymes, including superoxide dismutase (SOD), thioredoxin, glutathione peroxidase-1, heme oxygenase-1, and catalase [222]. Accordingly, experimental evidence has confirmed that resveratrol has anti-inflammatory, antioxidant, and antiapoptotic activities and a beneficial effect in preventing or slowing down a wide range of age-related diseases [223–225].

Resveratrol's neuroprotective effects appear to be mainly due to some of its physiological effects, such as stimulation of neurogenesis and microvessel formation as shown in aging rats [226]; stimulation of *β*-amyloid peptide clearance as shown in a transgenic AD mouse model [227]; inhibition of neuroinflammation, as shown in the inflammatory response in a mouse model of cerebral amyloid deposition [228]; and finally the reduction of mitochondrial oxidative stress as shown* in vitro* in neuroblastoma cells [229] and* in vivo* in the age-related cognitive dysfunction in old rats [230]. The neuroprotective effects of resveratrol dietary supplement were evaluated on the expression of markers for inflammation, oxidative damage, and cellular senescence in primary trabecular meshwork cells exposed to chronic oxidative stress. Results showed that resveratrol prevented the production of intracellular reactive oxygen species (ROS) and inflammatory markers (such as IL-1-alpha, IL-6, IL-8, and ELAM-1) [231]. In another study, the effects of the neuroprotective agents riluzole and resveratrol (each with different neuroprotection mechanisms), when administered alone or in combination, were evaluated on the survival of RGC in a rat model of glaucoma. Results indicated that RGCs were significantly preserved in all treatment groups compared to vehicle-only treated control animals and that (as expected) the association of the two neuroprotective agents gave better results than each one alone [232]. In a mouse model of mechanical optic nerve injury, long-term diet supplementation with resveratrol has been shown to delay RGC dendrite remodeling and loss [233]. In another recent work, the hypotensive effect of topical* trans*-resveratrol was evaluated in rats with steroid-induced ocular hypertension [234]. The maximum hypotensive effect was obtained with a 0.2% concentration of resveratrol and was evident both in normotensive animals and even to a greater extent in the hypertensive ones. Such effect disappeared in the presence of subtype A*₁* adenosine receptor antagonist, which was then considered responsible for the effect itself, as already known by previous experimental data [235]. In another* in vivo *study, resveratrol has been shown to protect rat RCGs against retinal ischemia/reperfusion injury induced by high intraocular pressure. The protection of resveratrol in these experiments was associated with the downregulation of the expression levels of matrix metalloproteinase-9, inducible nitric oxide, and heme oxygenase-1 [236].

## 14. Rutin

Rutin (also known as vitamin P or rutoside) is a flavonol glycoside found in many plants and fruits [237], resulting from the combination of the flavonol quercetin and the disaccharide rutinose. Indeed, upon oral administration, the disaccharide is cleaved, and quercetin is liberated, so that rutin's anti-inflammatory actions appear to be due to the quercetin-mediated effects via the inhibition of the proinflammatory chain triggered by TNF-*α*-induced NF-*κ*B activation [238].

Rutin has been shown to possess multiple pharmacological activities [239]. Two recent clinical studies have shown that oral administration of a food supplement containing rutin potentiates the hypotonizing effects of pharmacological treatments. It contributes to a better control and a further, although small, reduction of the IOP in POAG patients [38, 240]. Another clinical study showed that oral treatment with an association of forskolin and rutin can blunt the IOP spikes and avoid the damage that may occur after Nd:YAG laser iridotomy for the prevention of primary closed-angle glaucoma [241]. Furthermore, the association of forskolin and rutin has been shown to improve the symptoms of discomfort in glaucomatous patients suffering from dry eye induced by the long-term use of eye drops preserved with BAK [242].

Concerning the neuroprotective activity of rutin, different mechanisms have been suggested using both* in vivo* and* in vitro* models of neurodegeneration [243–245]. These include the reduction of possibly toxic nitric oxide levels, the inhibition of apoptotic triggers, and the upregulation of neurotrophic factors. In rat pheochromocytoma cells, rutin modulated several neuroprotective genes, including tyrosine hydroxylase, and was able to suppress caspase-3 activity [246]. Under hypoxic and glutamate stress conditions, rutin significantly increased the survival rate of neonatal rat RGCs by inhibiting the induction of proapoptotic caspase-3 and calpain [247]. The neuroprotective effects of rutin on the diabetic rat retina have been shown, likely contributed by rutin decreasing both activity and expression of caspase-3 and increasing the protein expression of the survival factor Bcl-2 [248].

Optimal levels of neurotrophic factors are necessary for normal neuronal functions such as synaptic activity and neuronal survival [249]. Rutin treatment significantly increased the levels of both BDNF and NGF in the retina of diabetic rats and activated BDNF and NGF gene expression in the hippocampus and brain of rodents [237, 250].

## 15. Conclusions

It is common knowledge that glaucoma is a slowly progressing neurodegenerative disease, in which RGCs are primarily affected. Several pathogenic mechanisms appear to be involved in glaucoma disease such as IOP elevation, ischemia/reperfusion, oxidative stress, neurotrophic growth factor deprivation, activation of autoimmunity, and glutamate neurotoxicity. Even if IOP control remains the gold standard for the pharmacological approach, it is clear from emerging research, as described in this review, that several molecules may interfere with the process leading to POAG progression, protecting RGC and preventing or at least delaying their apoptotic death, independently of IOP control.

Two main mechanisms emerge as critical targets for neuroprotection: (i) those linked to cAMP and (ii) those controlling oxidative stress and mitochondrial dysfunction.

cAMP is a second messenger involved in many different cellular pathways. It is pivotal in controlling AH secretion/reabsorption [251] and in regulating neurotrophin gene expression for RGC survival [252]. In fact, hypotonizing drugs such as alpha-agonists and beta-blockers plus forskolin and melatonin (although with different mechanisms) all impinge on cAMP production, neat AH secretion, and neuroprotection.

Oxidative stress is the main target of the other neuroprotective agents, including melatonin, described in this review.

Notably, the three compounds discussed here (namely, brimonidine, forskolin, and melatonin) all contribute at the same time to IOP control and neuroprotection, so that the association of these molecules together with current glaucoma therapies could lead to additional benefits for patients. Ideally, the best treatment for a glaucoma patient would include a hypotonizing agent together with a neuroprotective one spanning several different molecular neurotoxic mechanisms. Melatonin and/or forskolin appear to be good candidates to be associated with a classical hypotonizing drug, preferably brimonidine, considering its own neuroprotective effects.

Longer-term randomized clinical trials (RCT) are of course needed in order to prove this hypothesis, which opens an old, still unanswered problem about the role of RCT in neuroprotection. Indeed, a critical look at ways to take advantage of the current scientific and technological knowledge to run a reliable and affordable RCT could be an amenable and highly needed subject for a consensus conference and perhaps a future review paper.
